# Toward brain-inspired foundation model for EEG signal processing: our opinion

**DOI:** 10.3389/fnins.2024.1507654

**Published:** 2024-12-04

**Authors:** Suhan Cui, Dongwon Lee, Dong Wen

**Affiliations:** ^1^College of Information Science and Technology, The Pennsylvania State University, State College, PA, United States; ^2^School of Intelligence Science and Technology, University of Science and Technology Beijing, Beijing, China

**Keywords:** brain inspired computing, spiking neural network (SNN), brain computer interaction (BCI), foundation model, EEG signal analysis

## 1 Introduction

In recent years, AI and machine learning (ML) have revolutionized various fields of science and technology, with significant advancements in computer vision, natural language processing, and healthcare (Esteva et al., 2019). Despite this progress, applying these techniques to the analysis of electroencephalography (EEG) signals presents unique challenges due to the complex, non-stationary nature of brain activity. EEG is a critical tool for understanding brain dynamics in real-time, often employed in clinical diagnosis, cognitive neuroscience, and brain-computer interfaces (Schomer and Lopes da Silva, 2017). However, the noisy, high-dimensional nature of EEG signals makes it difficult to apply standard deep learning models effectively.

Foundation models, such as transformer-based architectures that have demonstrated unprecedented performance in fields like natural language processing and computer vision (Vaswani, 2017; Radford et al., 2021), hold great promise for addressing these challenges. These models are pre-trained on massive datasets and then fine-tuned for specific tasks, allowing for broad generalization and adaptability. However, their effectiveness in EEG analysis is limited, as they often lack mechanisms to capture the temporal precision and biological plausibility essential for accurately modeling brain signals (Roy et al., 2019).

One promising direction to overcome these limitations is the incorporation of brain-inspired algorithms into foundation models. Brain-inspired algorithms, such as spiking neural networks (SNNs), hierarchical temporal memory (HTM), and biologically plausible learning mechanisms like Hebbian learning, mimic the structure and function of neural processes (Schmidgall et al., 2024). These algorithms are designed to capture temporal and spatial dynamics more akin to those observed in actual brain networks. Incorporating these algorithms into foundation models could potentially bridge the gap between standard deep learning approaches and the dynamic, multi-dimensional nature of EEG signals.

Therefore, in this paper, we provide our opinions on how brain-inspired algorithms can be integrated with foundation models to enhance the analysis of EEG signals. We argue that by combining the scalability and generalizability of foundation models with the temporal specificity and biological plausibility of brain-inspired algorithms, this hybrid approach could address the current limitations in EEG signal processing. While the integration of these approaches poses significant technical challenges, their synergy could offer new pathways for more accurate and interpretable AI systems in neuroscience.

## 2 Current advances and challenges in EEG processing

In this section, we provide an overview of the current work in AI/ML based EEG signal processing, and discuss their limitations.

### 2.1 Advances in EEG signal processing

Recent advances in EEG signal processing have significantly improved our ability to extract meaningful insights from neural data. Traditionally, the feature-driven methods (Shoeibi et al., 2021) use specific features extracted from EEG signals to guide the analysis process. By leveraging the selected features through traditional machine learning classifiers, the models can uncover patterns in understanding EEG signals and brain activity. With the advancement of deep learning methods, researchers have started to utilize neural networks to better analyze the temporal and high dimensional EEG signals. Convolutional neural networks (CNNs) (Lawhern et al., 2018), recurrent neural networks (RNNs) (Li et al., 2022), Transformers Lee and Lee (2022) have shown promise in applications such as sleep stage classification, emotion recognition, and seizure detection (Craik et al., 2019). However, despite these advancements, limitations still remain in terms of both the data and model.

### 2.2 Challenges specific to EEG signal

EEG data present unique challenges that hinder the performance of conventional machine learning and deep learning models. First, EEG signals are highly non-stationary, meaning that the statistical properties of the signals vary over time and across individuals (Roy et al., 2019). This non-stationarity makes it difficult to develop models that can generalize well across different sessions, subjects, and experimental conditions.

Furthermore, EEG is known for its low signal-to-noise ratio (SNR), as the recorded signals are often contaminated by artifacts such as muscle movements, eye blinks, and electrical interference (Islam et al., 2016). Removing these artifacts without losing valuable information from the underlying brain activity remains a significant challenge.

Another major challenge is the spatial and temporal resolution of EEG. While EEG provides excellent temporal resolution (on the order of milliseconds), its spatial resolution is relatively poor compared to other neuroimaging techniques like fMRI or MEG. This limitation makes it difficult to localize brain activity with high precision, which in turn affects the performance of algorithms attempting to decode complex cognitive states or neural signatures (Buzsaki, 2019).

### 2.3 Limitations of foundation models in EEG processing

Foundation models, which have achieved remarkable success in natural language processing and computer vision, have not yet demonstrated comparable performance in EEG signal processing. One fundamental limitation is the lack of alignment between the structure of these models and the temporal dynamics of EEG signals. Foundation models, such as Transformers (Vaswani, 2017), are designed to capture relationships within structured data, such as words in a sentence or pixels in an image, but EEG signals are far more irregular and dynamic. Unlike text or image data, which exhibit consistent patterns that foundation models can exploit through attention mechanisms, EEG signals require a model that can handle continuous temporal fluctuations and low signal amplitudes (Craik et al., 2019).

Additionally, foundation models require extensive amounts of training data; however, EEG datasets tend to be limited and imbalanced due to the high cost and time-intensive nature of data collection (Kher, 2020; Zheng and Lu, 2015). Pretraining a foundation model for EEG signals demands the aggregation of diverse datasets from multiple sources, which presents significant challenges for the training process. Although techniques like data augmentation and transfer learning have been proposed to address these limitations (Jayaram et al., 2016), they remain insufficient for enabling foundation models to generalize effectively to new EEG datasets.

Finally, the depth and scale of foundation models inherently cause higher latency due to the time taken to process multiple layers and large numbers of parameters. In real-time EEG applications (Müller et al., 2008), even small delays in data processing can degrade performance significantly. Thus, the large size and complexity of foundation models can conflict with the need for low-latency performance for EEG signal processing.

## 3 Potential of brain-inspired algorithms for EEG processing

Brain-inspired algorithms represent a cutting-edge approach within artificial intelligence (AI), where computational models are designed to emulate the biological processes and mechanisms of the brain (Chen et al., 2022). This approach aims to bridge the gap between the relatively rigid frameworks of traditional AI models and the flexible, adaptive, and efficient nature of biological cognition. In this section, we analyze the current brain-inspired algorithms and discuss their potential to improve the foundation models for EEG signal processing.

### 3.1 Spiking neural networks

Spiking neural networks (SNNs) offer a promising approach to overcoming some of the limitations of foundation models for EEG processing. Unlike conventional neural networks, SNNs mimic the brain's natural processing by incorporating time-dependent spiking activity, which makes them better suited to handling the temporal dynamics of EEG signals (Maass, 1997). SNNs process information as sequences of spikes, which enables them to capture the temporal structure of neural data more effectively than traditional models that treat data in a continuous manner. Additionally, SNNs are event-driven, meaning that computation only occurs when relevant spikes are received, potentially leading to more efficient processing of EEG data compared to standard deep learning models that rely on continuous input. Recent studies have demonstrated the advantages of SNNs in various EEG applications (Choi, 2024).

### 3.2 Hierarchical temporal memory

Hierarchical Temporal Memory (HTM), inspired by the neocortical structure of the brain, represents another brain inspired model that shows potential in EEG data processing. HTM is particularly adept at learning sequences and detecting temporal patterns, which aligns with the nature of EEG signals (Hawkins and Ahmad, 2016). Also, HTM networks operate on principles of temporal memory and spatial pooling, allowing them to detect and predict complex sequences from noisy, incomplete data—key characteristics of EEG signals. Due to these properties of HTM, it has been widely applied to time series analysis (Struye and Latré, 2020; Wu et al., 2018).

### 3.3 Hebbian learning

Besides the neural network models, the brain-inspired learning rules such as Hebbian learning also presents potential for improving the EEG signal processing. Hebbian learning, which is based on the principle that “cells that fire together wire together,” allows networks to self-organize based on the correlations between neuron activations (Markram et al., 1997). In EEG processing, this could lead to more adaptive models that are better aligned with the plastic nature of the brain, enabling models to learn representations that are more flexible and robust to noise and variability. Also, such learning rules could be combined with brain-inspired neural networks, improving their performance in various applications (Uleru et al., 2022; Long and Gupta, 2008; Kozdon and Bentley, 2018).

## 4 Our opinion: combining brain-inspired algorithms with foundation models

**Figure 1:**
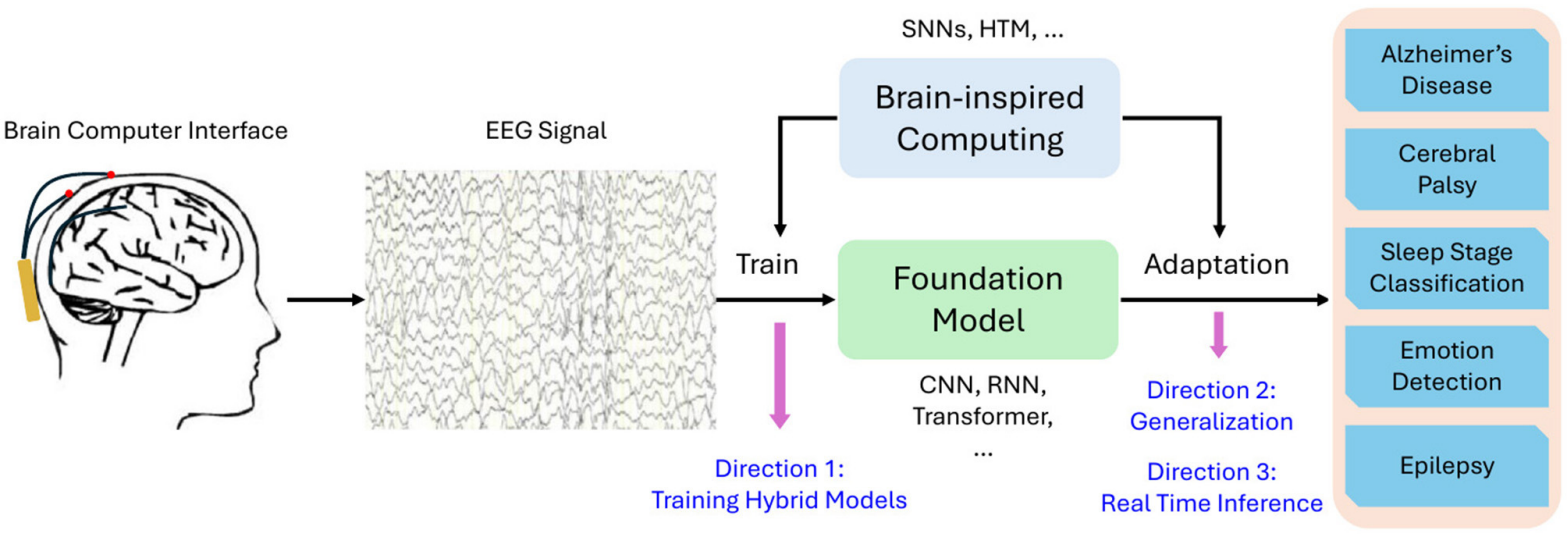
Schematic representation of hybrid brain-inspired foundation models for EEG signal analysis.

Based on the analysis above, we propose one promising direction for improving EEG signal processing. That is the integration of brain-inspired algorithms with foundation models to create hybrid architectures. Hybrid models could combine the strengths of foundation models–such as their scalability and ability to generalize across tasks–with the temporal and spatial specificity of brain-inspired algorithms. We schematically show this idea in Figure 1.

### 4.1 Overview

Specifically, SNNs or HTM could be incorporated during both the training and adaptation phases of foundation models. The optimization techniques inherent in SNNs and HTM could enhance the training of foundation models, enabling them to learn more effective representations of EEG signals. Hebbian learning could also be applied to train the foundation models which further enhances the representation. Additionally, when adapting foundation models to downstream tasks such as Alzheimer's disease diagnosis and emotion detection, the dynamic properties of SNNs could be leveraged for flexible, task-specific adaptation, allowing the model to more effectively capture the nuances of each particular application. Studies have begun to explore such hybrid approaches. For instance, Kheradpisheh et al. (2018) proposed a hybrid architecture that combines SNNs with deep learning for image classification tasks, and their approach could be adapted for EEG signal. This integration could help overcome the temporal limitations of foundation models, while still allowing them to leverage large-scale training data and sophisticated attention mechanisms. Moreover, combining foundation models with more lightweight, brain-inspired algorithms could offer a solution that balances performance with the real-time constraints of EEG processing.

### 4.2 Strengths

Therefore, hybrid models offer substantial opportunities for enhancing EEG processing. By incorporating brain-inspired components, these systems could potentially offer a more accurate representation of brain activity, enhancing performance in tasks such as cognitive state classification, seizure detection, and the diagnosis of neurodegenerative diseases. As research in this area progresses, it may lead to more advanced models that combine biological fidelity with the computational power of modern foundation models, enabling more robust and interpretable solutions for neural signal analysis.

### 4.3 Limitations

However, implementing hybrid systems poses several challenges. First, designing such hybrid systems requires efficient communication between different model components, as well as the balance between biological plausibility and computational efficiency. Moreover, training hybrid models can be difficult, as integrating spiking networks with conventional deep learning models requires novel optimization techniques and specialized hardware. Furthermore, the EEG based applications often require real time processing, so how to employ the pre-trained foundation model in real-time systems remains a challenge. Lastly, since EEG datasets are normally small and task specific, pre-training over multi-sourced EEG datasets might also adds to the training challenges of foundation models.

## 5 Future directions

We here summarize our opinions into several future directions for the integration of brain-inspired algorithms with foundation models for EEG signal processing. Specifically, future research should focus on several key areas:

- **Improved training techniques for hybrid models:** New training methodologies are needed to enable the integration of spiking networks with traditional deep learning layers and to effectively manage diverse pretraining EEG datasets. This challenge can be addressed from both software and hardware perspectives. For example, applying surrogate gradient methods (Neftci et al., 2019) can optimize the hybrid model in a gradient-based manner, while neuromorphic hardware optimizations (Davies et al., 2018), can improve the training efficiency for SNNs.
- **Scalability and generalization:** For practical use in clinical applications, hybrid models must be scalable to large, diverse EEG datasets while maintaining performance across a wide range of neurophysiological tasks. Transfer learning approaches that enable pre-trained foundation models to adapt to smaller EEG datasets could provide a pathway toward achieving better generalization.
- **Real-time applications:** Hybrid models need to be optimized for real-time processing, particularly for BCIs and neurofeedback applications, where low-latency are essential (Sharma and Meena, 2024). Future studies should explore novel architectures and neuromorphic hardware that allow for efficient online adaptation to incoming EEG data.

## 6 Conclusion

In conclusion, our opinion for this paper is that incorporating brain-inspired algorithms into foundation models offers a promising path forward for enhancing EEG signal processing. Brain-inspired approaches align more naturally with the temporal and noisy characteristics of EEG data. However, challenges remain in optimizing these hybrid systems for large-scale, real-time applications. Future research should focus on developing advanced training techniques, ensuring scalability and generalization, and enabling real-time performance. With further advancements, these hybrid foundation models could significantly improve the accuracy and usability of AI-driven EEG analysis.
